# Deep learning for sex classification in resting-state and task functional brain networks from the UK Biobank

**DOI:** 10.1016/j.neuroimage.2021.118409

**Published:** 2021-11-01

**Authors:** Matthew Leming, John Suckling

**Affiliations:** Department of Psychiatry, University of Cambridge, Cambridge, Cambridgeshire CB2 0SZ, UK

## Abstract

Classification of whole-brain functional connectivity MRI data with convolutional neural networks (CNNs) has shown promise, but the complexity of these models impedes understanding of which aspects of brain activity contribute to classification. While visualization techniques have been developed to interpret CNNs, bias inherent in the method of encoding abstract input data, as well as the natural variance of deep learning models, detract from the accuracy of these techniques. We introduce a stochastic encoding method in an ensemble of CNNs to classify functional connectomes by sex. We applied our method to resting-state and task data from the UK BioBank, using two visualization techniques to measure the salience of three brain networks involved in task- and resting-states, and their interaction. To regress confounding factors such as head motion, age, and intracranial volume, we introduced a multivariate balancing algorithm to ensure equal distributions of such covariates between classes in our data. We achieved a final AUROC of 0.8459. We found that resting-state data classifies more accurately than task data, with the inner salience network playing the most important role of the three networks overall in classification of resting-state data and connections to the central executive network in task data.

## Highlights

• Applied deep learning to sex classification in UK BioBank fMRI connectomes.
• Deep learning classifies sex better in resting-state than in task fMRI.
• Algorithm to balance out multiple confounds from an fMRI dataset.
• Adapted two deep learning visualization methods to fMRI connectome classification.
• Analyzed role of three brain a priori networks in sex classification.

## 1 Introduction

In recent years, neural networks have proven to be a powerful tool for classification of 2D and 3D images (Karpathy, Fei-Fei, 2014, Krizhevsky, Sutskever, Hinton, 2012, Maturana, Scherer, 2015). Because of their wide applicability in representing data such as proteins and social networks, much work has been done on adapting neural networks to accept graphs (i.e., networks of nodes interconnected by weighted edges) as input for tasks including whole-graph classification, clustering, and node-wise classification (Bruna, Zaremba, Szlam, LeCun, 2014, Defferrard, Bresson, Vandergheynst, 2016, Hamilton, Ying, Leskovec, 2017, Hechtlinger, Chakravarti, Qin, 2017, Kipf, Welling, 2017, Nikolentzos, Meladianos, Tixier, Skianis, Vazirgiannis, 2017).

Convolutional neural networks (CNNs) adapted for graphs have potent applications in the classification of functional connectivity; a functional MRI reduced to a correlational matrix – effectively a graph – that measures the inter-regional relationships between the blood-oxygen-level-dependent (BOLD) signals in predefined anatomical brain areas. While there is no consensus in the neurophysiological interpretation of the resulting networks, certain features have been found to be robust markers of different mental states and disorders; for instance, the default mode network, a large-scale subnetwork within the parietal and frontal areas, has been found to be a marker of resting (task absent) functional connectivity (Raichle et al., 2001).

While other machine learning (ML) models have been developed for analyzing graph data (Jie, Zhang, Wee, Shen, 2013, Kriege, Johansson, Morris, 2019), they have often been designed to characterize general networks (such as social networks) rather than fixed-node matrix representations, and so are not ideal for functional connectomes. Additionally, with its utilization of powerful deep learning structures (Brown, Kawahara, Hamarneh, 2018, Kawahara, Brown, Miller, Booth, Chau, Grunau, Zwicker, Hamarneh, 2017), CNNs are among the most promising ML tools for the diagnosis and prognosis of neurological and mental health disorders using graph representations of the structure and function of the brain. Recent work in this area has included innovations in deep learning models themselves: Parisot et al. (2018) and Li et al. (2019) applied graph neural networks (Kipf and Welling, 2017) to classify autism spectrum disorder in resting-state and task fMRI. Kim and Ye (2020) applied the recently-proposed graph isomorphism network (Xu et al., 2018) to classify sex in the brain, while (Gadgil et al., 2020) proposed an original spatio-temporal graph convolution to predict age and gender in rs-fMRI.

Although they may be applied to classify such graphs, CNNs (and indeed, neural networks more generally) often face a problem with interpretability. Even if CNNs can classify data successfully, it is unknown which features of the input data make a disproportionate contribution in the process, and the model remains a “black box.” Knowledge of such features are especially necessary for biological applications in which the underlying mechanisms of the systems being classified are often of the greatest interest. To overcome the black box problem, a number of ways to visualize and quantify neural networks have been pioneered in recent years. These methods include activation maximization (Erhan et al., 2009), in which the data that maximally activates a hidden node is recorded, occlusion, in which the classification accuracy is measured when specific input data are systematically omitted from the process (Zeiler and Fergus, 2013), and saliency maps (Simonyan et al., 2014), later adapted into gradient class activation maps (Selvaraju et al., 2017), in which the derivative of the neural network with respect to input data is approximated displaying which parts of the input data effected the most change in the neural network.

The problem of encoding graphs persists in the application of CNNs. Kawahara et al. (2017) previously employed salience maps in classifying connectivity matrices, using cross-shaped filters in convolutions, to show which connections in the brain had the greatest effect on the resultant classification (thus encoding edge-to-edge connections) instead of square-shaped filters that are more typical for 2D image classification. In our previous work, we used vertical-filters with CNNs and class activation maps to classify functional connectomes (Leming et al., 2020).

While encoding based on the columns of a connectivity matrix is intuitively sound, given that it accounts for the edges connected to a particular node, it does in theory have three problems. First, the convolutions bias the output class activation maps; a highly salient single edge would also increase the salience of edges in its same row or column. Second, it is difficult to determine the veracity of saliency algorithms from biological data where the ground truth is unknown, and for single runs the algorithms may give spurious results (Kohavi, 1995), whereas they often indicate “visual saliency” for 2D images (i.e., areas of the image on which human subjects focus), which are straightforward to verify by a human observer. Because of the inconsistencies between ML models, the most robust solutions come from averaging salience maps found over a number of trained models (Leming, Gorríz, Suckling, 2020, Meenakshi, Jamison, Kuceyeski, Sabuncu, 2018). Third, convolving whole columns or rows with a single value (node) encodes a large amount of input data that scales with the size of the input matrix. This dilutes the relative contributions of single edges which may be essential in classification, and possibly leads to underfitting.

### 1.1 Network brain function across the sexes

Taken on their own, differences found between task-based and resting-state brain activations may be among the most robust discoveries of fMRI studies. The default mode network (DMN) has been consistently identified as a marker of resting-state (i.e. in the absence of a cognitively effortful task) connectomes since it was first described (Raichle et al., 2001). Other brain networks emblematic of particular tasks have been identified as well (Smith et al., 2009), including the dorsal and ventral attention networks (Corbetta, Shulman, 2002, Vossel, Geng, Fink, 2014), which are respectively concerned with voluntary focus on features and switches in attention or unexpected stimuli; i.e., the change between resting-state and task fMRI. As noted by Fox et al. (2005), when performing simple memory tasks, the response commonly observed is proportionally increased activity in certain frontal and parietal cortical regions (Cabeza, Nyberg, 2000, Corbetta, Shulman, 2002) and decreased activity in the posterior cingulate, medial and lateral parietal, and medial prefrontal cortex (Gusnard, Akbudak, Shulman, Raichle, 2001, Mazoyer, Zago, Mellet, Bricogne, Etard, Houdè, Crivello, Joliot, Petit, Tzourio-Mazoyer, 2001, McKiernan, Kaufman, Kucera-Thompson, Binder, 2003, Shulman, Fiez, Corbetta, Buckner, Miezin, Raichle, Petersen, 1997, Simpson, Snyder, Gusnard, Raichle, 2001), which form the default mode network. Fox et al. (2005) identified two widely distributed, anticorrelated networks in the brain that exist in the resting state, but intensify during tasks. Additionally, switches between the resting-state and task often involve transitions from the DMN to the central executive (CEN) and salience networks (Goulden et al., 2014). The CEN is the dominant network following suppression of the DMN when a cognitively demanding task is being performed (Fox et al., 2006), while the salience network is activated in a less task-specific manner and more in response to perceived cognitive, homeostatic, or emotional salience (Seeley et al., 2007), which may be brought on by pain, uncertainty, or emotional tasks. Effective connectivity studies with granger causality (Sridharan et al., 2008) and dynamic causal modeling (Goulden et al., 2014) have indicated that the DMN to CEN transition is modulated by the salience network.

Sex differences in brain networks, and more generally the functional processing of tasks, is an area of active scientific interest. But while functional imaging studies of the brain have often found differences between men and women, it is difficult to compare studies due to small sample sizes, differing analysis methods, different areas selected a priori for testing, and differences in particular tasks. Various task fMRI studies have found widely spread sex differences in the bilateral amygdala, hypothalamus, right cerebellum, and posterior and superior temporal sulcus in response to emotional and visuospatial processing (Hamann, Herman, Nolan, Wallen, 2004, Mackiewicz, Sarinopoulos, Cleven, Nitschke, 2006, Takahashi, Matsuura, Yahata, Koeda, Suhara, Okubo, 2006); right hemisphere activation in response to visuospatial tests (Gur et al., 2000); differing activations in the superior parietal lobule and the inferior frontal cortex in response to mental rotation tasks (Hugdahl et al., 2006); and limbic regions, prefrontal regions, visual cortex, the anterior cingulate gyrus, and the right subcallosal gyrus in response to emotional faces (Fischer, Sandblom, Herlitz, Fransson, Wright, Backman, 2004, Fusar-Poli, Placentino, Carletti, Landi, Allen, Surguladze, Benedetti, Abbamonte, Gasparotti, Barale, Perez, McGuire, Politi, 2009).

Three large sample-size neuroimaging studies that documented functional sex differences in resting-state fMRI in both developing (Gur, Gur, 2016, Tomasi, Volkow, 2011) and adult populations (Ritchie et al., 2018) have been conducted. These studies found higher local functional connectivity in women than in men, higher connectivity in the DMN in women, and lower connectivity in the sensorimotor cortices. However, unlike the emotional stimuli studies, there were no particularly localized differences in activation between the samples. This was possibly due to the higher variation of resting-state fMRI due to its unconstrained nature (Buckner, Krienen, Yeo, 2013, Elton, Gao, 2015).

The effects of sex on macro resting-state and task networks are still debated (Goldstone et al., 2016). Some studies (Agcaoglu, Miller, Mayer, Hugdahl, Calhoun, 2015, Liu, Stufflebeam, Sepulcre, Hedden, Buckner, 2009) have found that sex modulates the lateralization of resting-state networks, while other studies have reported only a small (Bluhm, Osuch, Lanius, Boksman, Neufeld, Théberge, Williamson, 2008, Lopez-Larson, Anderson, Ferguson, Yurgelun-Todd, 2011) or non-significant effect (Nielsen, Zielinski, Ferguson, Lainhart, Anderson, 2013, Weissman-Fogel, Moayedi, Taylor, Pope, Davis, 2010). Network-level sex differences in task fMRI indicate that men and women process tasks differently. Adolescent females have been reported as having higher functional connectivity in the DMN and fronto-parietal networks during a self-referential processing task (Alarcón et al., 2018). Analysis of canonical networks in task fMRI, although not able to draw substantial conclusions on the roles of the networks in different tasks, found that tasks involving fluid intelligence were the most discriminative for sex (Greene et al., 2018). These studies would suggest that men and women process tasks differently. However, they have not been validated on larger datasets.

### 1.2 Machine learning for sex classification

When classifying between sexes, past ML studies using methods ranging from support vector machines (SVMs) to CNNs, have achieved classification accuracies between 65% and 87% (Casanova, Whitlow, Wagner, Espeland, Maldjian, 2012, Gur, Gur, 2016, Satterthwaite, Wolf, Roalf, Ruparel, Erus, Vandekar, Gennatas, Elliott, Smith, Hakonarson, Verma, Davatzikos, Gur, Gur, 2015, Zhang, Groen, Mennes, Greven, Buitelaar, Rommelse, 2018), depending on the dataset and methods used. In Leming et al. (2020), we performed a classification by sex of functional connectomes acquired at multiple sites using a CNN with vertical filters, with a final area under the receiver operating characteristic curve (AUROC) of 0.7680, including an AUROC of 0.8295 with single-site, UK BioBank data. Recent studies in sex classification have highlighted the complexities of cross-sample classification and the need for class balancing. Using an SVM on two rs-fcMRI samples from the Human Connectome Project (HCP) and one from the 1000Brains study, Weis et al. (2020) achieved 75.1

The objective of this article is not only to utilize CNNs to classify functional connectomes, but explain the classification performance in terms of those edges and subnetworks that are most salient. To do so, we introduce a stochastic deep learning model that allows for the consideration of each edge in a network independently without overfitting, presenting robust results by training and combining many such models in the ensemble framework first proposed in Leming et al. (2020). Convolutions with random samples of edges allow for the consideration of each edge independently without overfitting to one particular edge (which would be the case with fully-connected neural networks). However, in training many such models and averaging their outputs, this scrambling does not suffer from the issue of spatial biases seen in class activation maps with vertical convolutions (an effect that may be observed in Leming et al. (2020)).

In this paper, we used CNNs and utilized big data to characterize sex differences in connectomic representations of resting-state and task fMRI (in UK Biobank data, a faces/shapes “emotion” task Barch, Burgess, Harms, Petersen, Schlaggar, Corbetta, Glasser, Curtiss, Dixit, Feldt, Nolan, Bryant, Hartley, Footer, Bjork, Poldrack, Smith, Johansen-Berg, Snyder, Van Essen, Consortium., 2013, Hariri, Tessitore, Mattay, Fera, Weinberger, 2002) with a focus on the DMN, the salience network, and the CEN. We trained our CNNs to classify sex in an extremely large dataset: 16,970 fMRI acquisitions from the UK BioBank, decomposed into multi-wavelet-frequency functional connectivity matrices (Patel, Bullmore, 2016, Patel, Kundu, Rubinov, Jones, Vertes, Ersche, Suckling, Bullmore, 2014). To eliminate the effects of factors such as age, head motion, and intracranial volume, we also detail a multivariate class balancing scheme that ensured equal distributions of these factors within statistical significance. We evaluated performance with the average AUROC, a standard measure of accuracy in ML, across 300 models in an ensemble scheme. We then used guided gradient class activation mapping (Grad-CAM) (Selvaraju et al., 2017) and occlusion (Zeiler and Fergus, 2013) of individual brain networks to evaluate the salience of each edge within and connecting brain networks, comparing their relative salience within the model.

## 2 Methods

### 2.1 Pre-processing

#### 2.1.1 Data acquisition and pre-processing

The dataset was fMRI data from the UK Biobank, which included both resting-state and task data from a faces/shapes “emotion” task (Barch, Burgess, Harms, Petersen, Schlaggar, Corbetta, Glasser, Curtiss, Dixit, Feldt, Nolan, Bryant, Hartley, Footer, Bjork, Poldrack, Smith, Johansen-Berg, Snyder, Van Essen, Consortium., 2013, Hariri, Tessitore, Mattay, Fera, Weinberger, 2002). Details of the acquisition parameters are given elsewhere (Ritchie et al., 2018).

Pre-processing was completed with the fMRI Signal Processing Toolbox (SPT; www.brainwavelet.org). Following initial identification of the brain parenchyma, and affine registration of the 4D sequence to the mean of the sequence, head motion correction was accomplished using SpeedyPP version 2.0. This process utilized AFNI tools and wavelet despiking (Patel, Bullmore, 2016, Patel, Kundu, Rubinov, Jones, Vertes, Ersche, Suckling, Bullmore, 2014), with low- and high-bandpass filters of 0.01Hz and 0.1Hz, respectively, in addition to motion and motion derivative regression. Three motion indicators measured with tools in FSL (FSL motion outliers and FAST; fsl.fmrib.ox.ac.uk/fsl) were recorded that were later applied in class balancing: framewise displacement, spike percentage values, and DVARs. Thus, even if motion correction were imperfect, each dataset would have the same distribution of motion values in either class.

Time-series at each voxel in the brain were wavelet despiked to remove transient signals, and then functional and structural datasets were registered to Montreal Neurological Institute (MNI) space and parcellated using the 116-area automated anatomical labeling (AAL) template, including subcortical regions (Tzourio-Mazoyer et al., 2002), that defined the nodes of the graph.

The average BOLD signal from each parcel was decomposed by wavelet transform in to three frequency bands: 0.05-0.1 Hz, 0.03-0.05 Hz, and 0.01-0.03 Hz. In each frequency band, separately for each of N datasets, the correlation of the 3 wavelet coefficients between 116 parcels estimated the edge weights, resulting in N N×3×116×116 symmetric connectivity matrices.

Intracranial volume was estimated from structural images with FSL FAST.

Pre-processing was accomplished on a server cluster over a period of several weeks. Due to the volume of datasets, individualized quality control was not possible. From beginning to end, 34.8% of datasets failed the parcellation/wavelet correlation stages and were rejected from further analysis.

#### 2.1.2 Dataset balancing of confounding factors

When viewed across the full dataset, there were clear differences in the distributions of covariates when stratifying data by both sex and resting-state/task. sex differences in intracranial volume are well-documented (Ruigrok et al., 2014), and differences in head motion in resting-state and task datasets were also observed. To address these confounding factors, we implemented an algorithm to balance the datasets such that confounding factors, if successfully measured, were not statistically different between groups. This algorithm first required continuous covariates (such as mean framewise displacement, intracranial volume, and age) to be discretized such that values within a given range are placed into “bins”, with each bin covering an equal span of values. Covariates such as collection were already discrete.

The algorithm curated a subset of the total dataset such that a datapoint from class A within bins $b_{1},b_{2},\ldots b_{n}$ had a corresponding datapoint within the same multivariate bins from class B that was also within the bins $b_{1},b_{2},\ldots b_{n}$. In effect, and bearing in mind that males have larger average intracranial volumes, females with smaller intracranial volumes and males with larger intracranial volumes were used less often in the training set, while males with smaller intracranial volumes and females with larger intracranial volumes were more likely to be included in a particular sampling. There is a trade off between the size of individual bins and the size of the dataset, since larger bins are naturally more inclusive, but allow for more variation in the distribution of covariates. Thus, the minimum number of bins was used such that it would not reject the null hypothesis with a nonparametric Mann-Whitney U-test with p>.10, with the algorithm iteratively increasing bin count on each confounding factor and applying the Mann-Whitney U-test until this threshold was achieved. We balanced by age, mean framewise displacement (MFD), intracranial volume (ICV), mean DVARs, and mean spike percentage.

This algorithm was applied twice to our data. The first balanced men and women. This scheme forced a 1:1 ratio between sexes, with distributions of respective covariates maintained. Data was then balanced by resting-state and task, though no ratios were forced. This left four divisions in the data: resting-state and task, men and women, with approximately equal distributions of confounding factors.

### 2.2 Machine learning

We classified functional data from the UK BioBank by sex. Because classification of UK BioBank rest/task data achieved near-perfect accuracy in Leming et al. (2020), we chose not to repeat this analysis. Here, the focus was on the relative classification accuracy of task data and resting-state data when classifying by sex.

#### 2.2.1 Model structure

**Fig. 1:**
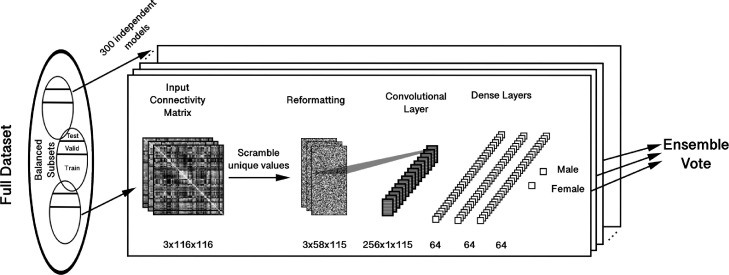
In this model, matrices are encoded by random scrambling prior to being fed into a single convolutional layer, followed by three dense layers. In between each layer is a batch normalization and rectified linear unit (ReLU) layer, with 50 percent dropout in between the dense layers. Our training scheme trains 300 such models, each with its unique scrambling order, independently on a class- and covariate-balanced subset of the whole dataset, then combines votes for datapoints appearing in overlapping test sets into a final ensemble vote.

The deep learning model was an ensemble of scrambled CNNs implemented in Keras with a Tensorflow backend. The architecture is shown in Fig. 1. We first randomly permuted the unique values (nodes) of the connectivity matrices, preserving the permutation order across wavelet frequency bands. These matrices were then input to a CNN with 256 filters of shape 1×58×1. This convolved 58×3 random values of the matrix which was then fed into three dense layers, each with 64 hidden units, with batch normalization layers, rectified linear unit (ReLU), and 0.5 dropout between them. Finally, the data was binary classified through a softmax layer.

The scrambling procedure removes any spatial priors in the encoding of data and is implemented to remove spatial biases from the visualizations. This would seemingly take away the purpose of convolutions in the model, but we maintain it for two reasons. First, it allows for the use of the internal analysis method we previously proposed in Leming et al. (2020), which may be applied in future studies, and generally makes it more comparable to that work. Second, because of the weight-sharing effects of convolutions, which reduce the total number of trainable parameters, it acts as a regularization method that builds on the dropout and batch normalization layers, which is appropriate for small training samples that may be prone to overfitting (Kukačka, Golkov, Cremers, 2017, Ott, Linstead, LaHaye, Baldi, 2020). To further validate the model, we additionally compare it to the performance of an ensemble of fully-connected neural networks lacking the convolutional layers.

#### 2.2.2 Training

The data were separated into training, validation, and test sets, with an approximate ratio of 4:1:1. We trained 300 CNN models on random class-balanced subsamples of the whole dataset, using an Adam optimizer with a categorical cross-entropy loss function; otherwise, Keras default settings were used. Each model was trained for 100 epochs (cycles through the training set), and the epoch with the highest validation accuracy was selected. CNN performance was reported on the test set. These 300 models with their respective test set classifications were then unified in an ensemble model. The output classification of a dataset appearing in $\frac{n}{300}$ models was averaged across n models. As models were trained independently of one another and only the accuracies reported on their respective test sets were averaged, no mixing occurred between the test and training sets. Thus, datasets were not counted more than once when measuring the final accuracy of the ensemble models, reported as AUROCs. In total, 14,683 datasets were used at least once in the test sets, comprising 86.5% of the overall dataset.

### 2.3 Visualization of machine learning results

We used two different ML visualization methods to assess the role of three different, a priori brain networks in the sex classification of resting-state and task data.

#### 2.3.1 Brain network encoding

**Fig. 2:**
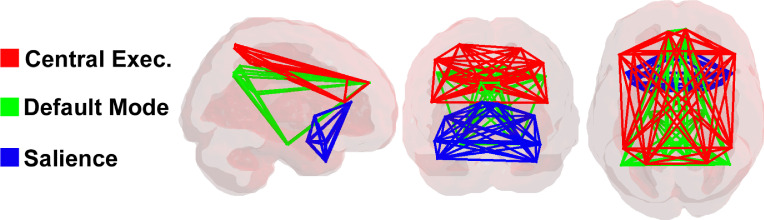
A 3d display of the three networks analyzed in this paper, in the AAL parcellation. Green: default mode network; blue: salience network; red: central executive network. Each network is comprised of ten distinct brain regions. (For interpretation of the references to colour in this figure legend, the reader is referred to the web version of this article.)

To assess the role of the DMN, CEN, and salience network in classification, we selected representative nodes from the AAL parcellation (named in Fig. 5), referring to prior network descriptions (Mulders et al., 2015). Each network comprised 10 distinct nodes. The DMN was characterized by a combination of the medial frontal gyrus, posterior cingulum, parahippocampus, precuneus, subgenual anterior cingulate cortex, and inferior parietal lobe, the CEN by the bilateral middle frontal lobe, frontal interior triangularis, frontal superior medial, and the superior and inferior parietal lobe, and the salience network by the bilateral insula, anterior cingulum, amygdala, and the middle and superior temporal pole (Fig. 2).

For both of the analysis methods described below, we isolated edges making up these networks in two different ways: first, by exclusively selecting edges within the network; i.e. edges connecting two nodes of a given network (comprising $\frac{10\times (10-1)}{2}=45$ unique edges); and second, all edges both within and connecting to a network, by selecting those edges that connect to at least one other node (comprising $10\times \left(116-1\right)-\frac{10\times (10-1)}{2}=1105$ unique edges). Thus, for each analysis method, two sets of results are presented: one for the sets of edges within a network, and the other for all edges connected to a network.

#### 2.3.2 Gradient class activation maps

We applied the Grad-CAM algorithm (Erhan, Bengio, Courville, Vincent, 2009, Kotikalapudi, contributors, Selvaraju, Cogswell, Das, Vedantam, Parikh, Batra, 2017) to find class activation maps (CAMs) for each dataset in each CNN model. These are invaluable visualizations that may be employed to determine which areas of input data a deep learning model focuses on in making its classification decision. Grad-CAM is an extension of the general salience algorithm (Simonyan et al., 2014). In its simplest form, salience is obtained by taking the derivative (approximated as a first-order Taylor expansion) of a particular deep learning model with respect to a particular input image. In studies of 2D images, CAMs are able to distinguish between different objects within a single image belonging to different classes (Selvaraju et al., 2017); for example, in a multiclass classifier of a picture of a cat and a dog, taking an image with respect to class 0 would highlight the cat, while taking the same image with respect to class 1 would highlight the dog. Grad-CAM extends this by making CAMs applicable to a variety of CNNs, including those that use fully-connected deep layers, as used here. In recent years, they have been applied to various deep learning MRI classification tasks (Lee, Lee, Lee, Park, Yoon, 2018, Zhang, Hong, McClement, Oladosu, Pridham, Slaney, 2021).

We derived CAMs from each independent stochastic CNN with respect to both class 0 (females) and class 1 (males) across three wavelet bands and averaged these across the 300 models, producing a single 116×116 CAM for each fMRI dataset in the ensemble models. The total distribution for CAM values within and connecting to each particular brain network was then compared to every other CAM value. Due to the extremely large number of values, distributional differences were measured by Cohen’s d (effect size), rather than statistical significance.

#### 2.3.3 Occlusion

In separate sex classification models, we occluded half of the edges for each model in the ensemble and trained on the occluded data. This was inspired by photographic image occlusion (Zeiler and Fergus, 2013) which deliberately excludes portions of data and measures relative classification accuracy with the occluded data as a means of detecting salient areas. The importance of the three brain networks to the classification was tested by comparing the average AUROC of 300 models whose occluded edges were the edges making up the particular brain network, and 300 models for which brain networks were not occluded. We trained on each set using the same 300 model/ensemble scheme detailed above (see Fig. 6, top). The relative accuracies of these independent models, both on the complete dataset and for the resting-state and task fMRI data, were compared to understand the contributions of different networks to sex classification in both resting-state and task fMRI. In particular, we applied a nonparametric statistical test on the two sets of 300 AUROCs including and excluding a particular brain network, then reported the p-value of this test, corrected for multiple comparisons.

We trained, for each of the three networks, 300 models that included the given network and 300 excluding it, each with the two different encoding schemes (i.e. considering the edges only within a network and all edges connected to a network), for each of the three networks (DMN, CEN, and salience network). In total, we trained 2×2×3×300=3600 models for these occlusion tests.

## 3 Results

### 3.1 Datasets and pre-processing

#### 3.1.1 Dataset balancing

**Fig. 3:**
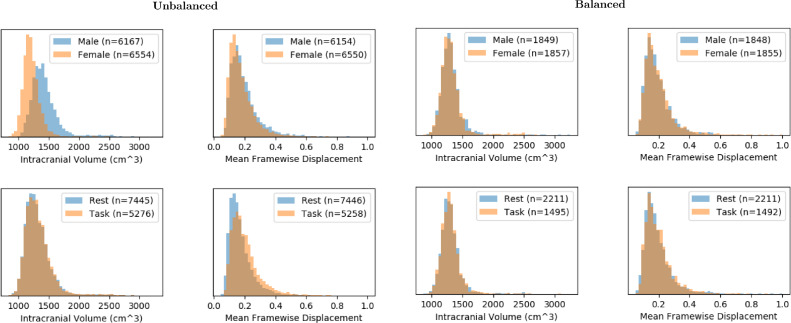
Histograms displaying distributions of random training sets with respect to mean FD and intracranial volumes, divided both by sex and resting-state/task, before and after the class balancing scheme.

The datasets displayed significant motion effects between groups, especially with regards to task- and resting-state differences, as well as significant differences in intracranial volumes between sexs (Fig. 3). The class balancing scheme selectively eliminated datasets such that each class had similar distributions across each covariate, as well as a 1:1 ratio of males to females. The same balancing procedure was also performed for resting-state and task data, with the original ratios present in the dataset maintained. Class balancing disincentivized the model from classifying based on confounding factors. The balanced class distributions can be seen at the bottom of Fig. 3.

### 3.2 Machine learning

#### 3.2.1 Model accuracy

We initially classified by sex balanced datasets with both resting-state and task fMRI. We used 300 independent CNNs that took as input randomly scrambled unique values of the input wavelet correlation matrices (Fig. 1) in a stratified cross-validation (Kohavi, 1995) scheme. The final results for the 300 models are given in Table 1 (top row) with an average AUROC of 0.8010 when assessing the CNNs independently. However, when all 300 models were aggregated into a single classification such that predictions for a particular dataset appearing across multiple independent models were averaged into a single value (Fig. 1), the AUROC was 0.8459. The same scheme was run with 300 independent fully-connected neural networks, with the same structure as described above except lacking convolutional layers; the resulting AUROC was 0.8318.

**Table 1:** Ensemble and Mean AUROCS of all models. Distributions of each of these classification accuracies are shown in Fig. 6.

		All		Rest		Task	
		Ens.	Mean ± Std	Ens.	Mean ± Std	Ens.	Mean ± Std
Complete		0.8459	0.8010 ± 0.0164	0.8923	0.8504 ± 0.0184	0.7683	0.7207 ± 0.0285
Inner Edges Only							
CEN	Incl.	0.8380	0.7805 ± 0.0163	0.8844	0.8343 ± 0.0179	0.7609	0.7027 ± 0.0267
	Excl.	0.8386	0.7798 ± 0.0165	0.8825	0.8315 ± 0.0193	0.7641	0.7050 ± 0.0278
DMN	Incl.	0.8407	0.7804 ± 0.0160	0.8868	0.8336 ± 0.0189	0.7643	0.7018 ± 0.0289
	Excl.	0.8420	0.7804 ± 0.0168	0.8873	0.8334 ± 0.0182	0.7671	0.7030 ± 0.0301
SAL	Incl.	0.8388	0.7824 ± 0.0165	0.8860	0.8352 ± 0.0173	0.7600	0.7050 ± 0.0294
	Excl.	0.8392	0.7782 ± 0.0172	0.8853	0.8308 ± 0.0197	0.7631	0.7021 ± 0.0276
Connecting Edges							
CEN	Incl.	0.8406	0.7833 ± 0.0152	0.8872	0.8364 ± 0.0168	0.7624	0.7059 ± 0.0284
	Excl.	0.8287	0.7704 ± 0.0148	0.8738	0.8228 ± 0.0177	0.7544	0.6939 ± 0.0267
DMN	Incl.	0.8396	0.7801 ± 0.0171	0.8836	0.8337 ± 0.0187	0.7660	0.7020 ± 0.0304
	Excl.	0.8278	0.7712 ± 0.0162	0.8753	0.8246 ± 0.0196	0.7490	0.6929 ± 0.0273
SAL	Incl.	0.8397	0.7811 ± 0.0162	0.8875	0.8351 ± 0.0176	0.7619	0.7024 ± 0.0293
	Excl.	0.8321	0.7739 ± 0.0175	0.8853	0.8253 ± 0.0193	0.7631	0.6993 ± 0.0291

The ensemble model also classified sex in resting-state fMRI with an ensemble AUROC of 0.8923 and task fMRI with an AUROC of 0.7683, a difference of 0.1240. Full results are given in Table 1.

#### 3.2.2 Projection of ensemble upper limit

**Fig. 4:**
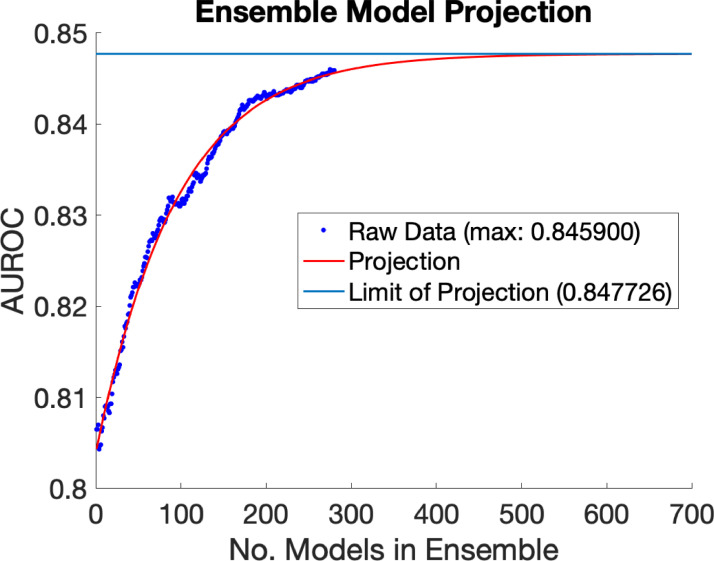
Sex classification AUROC across 1 - 300 independent CNNs included in the ensemble model. The raw data is plotted, as well as the projection of this trend using a logistics growth model ($y=\frac{a}{1+be^{-kx}},k>0)$, which assumes a hard upper limit (a) to the classification accuracy that can be achieved by simply increasing the number of models in the ensemble. The model predicts that simply adding more models to the ensemble beyond 300 achieves limited returns. The upper limit is 0.8477, with 95% confidence bounds between 0.8473 and 0.8481.

The upper predicted limit of AUROC in the limit of a large number of datasets, based on a logarithmic model, is shown in Fig. 4, and was found to be 0.8477.

**Fig. 5:**
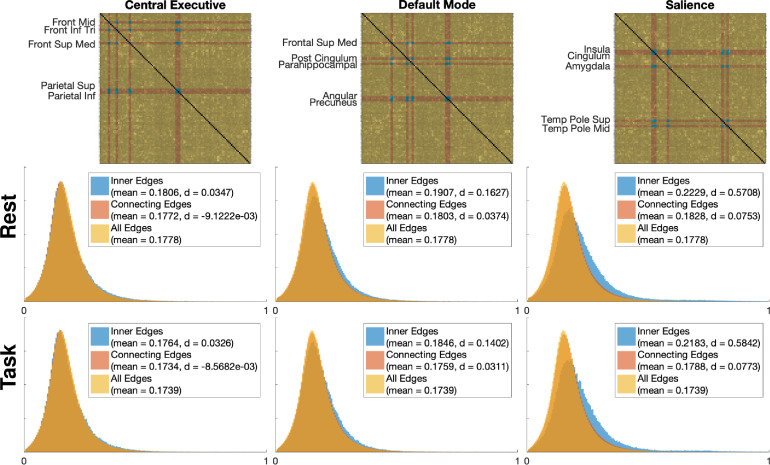
(Top) The averaged class activation maps (CAMs) across all subjects for the complete graph classification, with the three studied networks highlighted. Area names in the AAL atlas are given. (Bottom) Histograms of all inner and connecting CAM values of the three networks, both in resting-state and task subjects, compared to the overall distribution of CAM values. Because the large number of samples, we display the effect size (measured by Cohen’s d) of both inner and connecting edges compared to the CAM values of the rest of the edges.

**Fig. 6:**
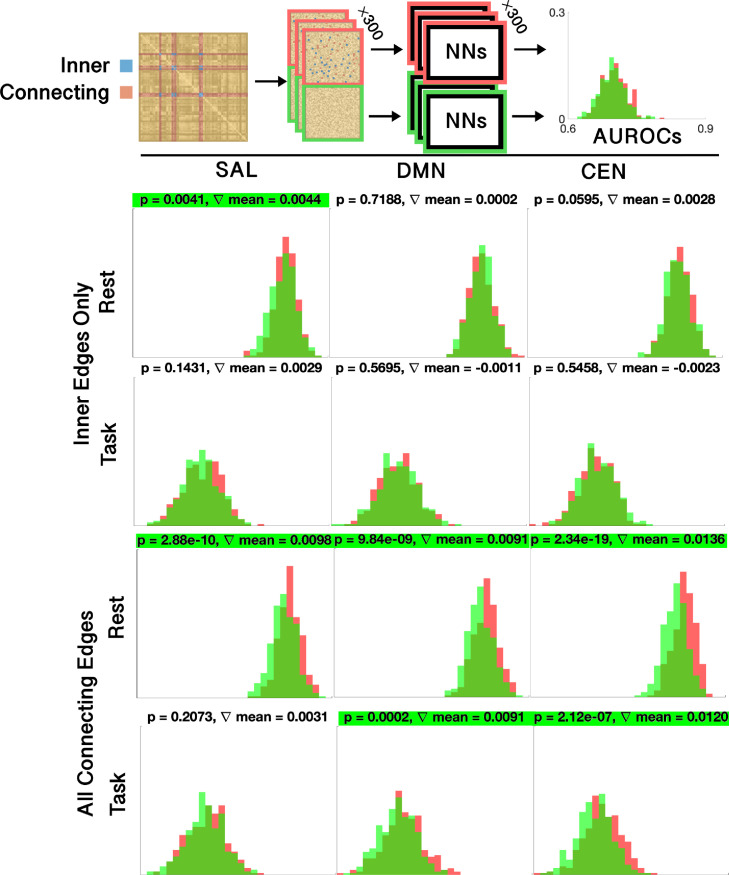
The effects of selective network occlusion on model accuracy. (Top) the process by which occlusion AUROCs are estimated; either all inner edges of a given network, or all edges connecting to a network, are selected. The network edges are then scrambled (see Fig. 1), and the selected edges are placed among one half of the scrambled edges, and in the other half left out. These two sets are then trained on 2×300 independent neural networks, and the resulting AUROCs are compared. (Bottom) The results. Considering only inner edges, the only statistically significant effect, after Bonferroni-Holmes correction, was the salience networks on resting-state data. Considering all connecting edges, all three networks had a significant effect on the classification of sex in resting-state data, while both the default mode network and, more strongly, the central executive network, appeared to have an effect in classification of task data. The nonparametric Mann-Whitney U-test was used to test for statistical significance. Final model means and ensemble results are shown in Table 1.

### 3.3 Visualization of machine learning results

#### 3.3.1 Gradient class activation maps

In total, 14,683 unique connectomes (comprised of both resting-state and task data) were classified by sex across 300 ensemble models. For each connectome, a single, 116×116 gradient class activation map (with 115×58 unique values) was derived that indicated the general importance each particular edge played into the classification of that participant.

The distribution of edge values from CAMs, both from edges within, and edges connected to the respective networks, are shown for task and resting-state data in Fig. 5. These distributions were compared to the relative distribution of all edges with aggregated values of 115×58 CAM values inside and outside of a priori networks, across 14,683 unique subjects, totalling just under 100 million values. Effect size were reported (as Cohen’s d; see Fig. 5).

The differences in CAM values of edges inside and outside the CEN were non-significant, while some effects were observed for the inner, but not connecting edges of the DMN. The largest effect was seen in the salience network, having an effect size of d>0.57 for task- and resting-state data separately. In CAMs overall, there were no significant differences between task- and resting-state edge values. This likely indicates that CAMs, while useful for showing which networks are important to the overall task of sex classification, are not useful for showing whether these networks were more or less important for resting-state or task data.

#### 3.3.2 Occlusion

Using the same dataset for the sex classification task, we compared the AUROCs of 300 independent models that classified a random half of the network’s edges. One set of 300 deliberately included the set of edges that constituted a network, and the other set of 300 excluded the same edges (Fig. 6, top). By comparing the AUROCs and finding a statistically significant difference, we could assess the influence of a particular network on the classification.

The relative classification AUROCs from the halves of edges that included edges both inside and connecting to the DMN, CEN, and salience networks, as well as models completely excluding them, are shown in Table 1, while Fig. 6 shows the distribution of AUROCs on 300 models including and excluding each network, for resting-state and task data.

When considering only the edges within a network (consisting of $\frac{45}{58*115}=0.67\%$ of total edges), modest losses in accuracy were observed (Fig. 6), but the only one that achieved statistical significance in a Mann-Whitney *U*-test after Bonferroni correction was the salience network classification in resting-state data. However, when excluding all edges connected to a network (consisting of $\frac{1105}{58*115}=16.57\%$ of total edges), a difference between resting-state and task data was observed: exclusion of all three networks led to a statistically significant (p<.05) decrease in AUROC for the classification of resting-state data, while the exclusion of the central executive and default mode, but not the salience networks, led to a statistically significant drop in AUROC for task, indicating less of a difference in the salience network between men and women in task fMRI, whereas such a difference was present in resting-state fMRI.

It may be the case that the set of edges connecting to a network contain redundant information to the edges within a network, explaining the more modest losses in accuracy when only within-network edges were excluded.

## 4 Discussion

### 4.1 Deep learning model

Because it is able to capture nonlinear patterns across complex datasets, deep learning is a powerful tool for characterizing biological data. However, because of interest in identifying patterns discovered by deep learning models, the interpretability of the model is just as important as performance, though it is far more difficult to quantify or even define (Doshi-Velez and Kim, 2017). The primary methodological contribution of this study is a model that captures the contributions of individual functional connections to fMRI deep learning classification, while the results of our data show that utilization of this model in the context of network neuroscience can shed light on between-sex differences in task- and resting-state brain networks.

Our model addresses an important problem unique to the issue of classifying graphs in CNNs, which is bias inherent in its encoding. There is no universal consensus on a method of encoding graphs for ML, though others have been proposed (Jie, Zhang, Wee, Shen, 2013, Kawahara, Brown, Miller, Booth, Chau, Grunau, Zwicker, Hamarneh, 2017, Kriege, Johansson, Morris, 2019, Leming, Gorríz, Suckling, 2020, Nikolentzos, Meladianos, Tixier, Skianis, Vazirgiannis, 2017, Tixier, Nikolentzos, Meladianos, Vazirgiannis, 2017). Whether encoding them randomly is the optimal method for classification accuracy is up for debate, though random encoding does avoid the problem of overfitting that is present in fully-connected neural networks, and it avoids bias in the output CAMs that results from using filters with a consistent shape. In other words, the use of linear filters results in whole rows or columns of a functional connectivity matrix being emphasized, rather than particular edges. Additionally, the training scheme helped to eliminate bias from the output CAMs. Simple averaging over a large number of models and stratified cross-validation (Kohavi, 1995) is just as important as the model architecture itself, because this allows for reduced bias from both confounding factors and natural variations in the output of nondeterministic deep learning models.

Respectively, the average AUROC for sex classification across all 300 models was 0.8010. When aggregated as an ensemble, the combined AUROC was 0.8459. This represents an improvement over our previous sex classification in Leming et al. (2020) which achieved an AUROC of 0.8295 on BioBank data (0.7683 across all datasets used) with a vertical-filter CNN balancing by only age and site. Nonetheless, due to the different balancing schemes, these two studies likely used a moderately different subset of the overall data, and so a direct comparison between the present stochastic and the previous vertical filter models in terms of accuracy is not strictly valid. Comparisons to other state-of-the-art ML studies are also not possible, since there is high variation in classification accuracy depending on how data was collected and processed (Leming et al., 2020), and few imaging studies have attempted a sex ML task on a dataset of this size.

Our training and multivariate class balancing schema, when combined, offered another uniquely important contribution. By only inputting to smaller, independent models subsets of data in which measurable confounding factors were balanced beyond any detectable statistical significance, we were able to effectively regress out any confounding factors that we were able to measure. However, by combining these subsets over a large number of independent models that were then combined in an ensemble, we were able to utilize the majority of the overall data in the end result without losing the effects of balancing. This allows us to be sure that our ML model utilized the majority of an imbalanced dataset, without achieving higher accuracy due to any confounding factors, particularly head motion and intracranial volume.

Although the balancing techniques employed prevented our model from gaining higher accuracy due to confounding factors such as age, head size, and motion, this does not necessarily mean that such differences had no influence. Class balancing does not prevent the model from internally separating data based on such factors and considering them (wholly or partially) independently. To illustrate this issue, we briefly present an analogy: consider a ML task in which pictures of different species of cat must be separated from pictures of different species of dog; such a model would likely identify generalized differences between each (e.g., the ear shape), while also containing internal representations of each type of cat and dog contained in the training set, relying on features unique to each individual species (e.g., stripes on a tiger). For instance, black fur color may be considered salient, even though it doesn’t necessarily help to separate cats from dogs, because it helps the dataset to subclassify both black panthers and black Labrador retrievers.

Nonetheless, we are confident that class balancing within a cross-validation scheme reduced the influence of differences in confounding factors. We emphasize the importance of each particular step in the ML classification to achieve the output CAMs. These are: (1) random encoding, rather than encoding based on rows or columns; (2) averaging the output of many ML models, as individual outputs have a stochastic element; and (3) stratified cross-validation using balanced subsets of the data across these models.

In this study, we did not show explicit tests for differences with respect to different confounding factors beyond the visual aid in Fig. 3. The proposed algorithm designed uses a balancing scheme that itself is based on iteratively testing p-values, and so testing differences with respect to these would just be a validation of the correctness of the code used. Furthermore, classification on unbalanced datasets was not undertaken in the study, since testing unbalanced datasets in a deep learning context would also have very little effect on our results: the unbalanced dataset would very likely result in higher classification accuracy because, with confounding factors to aid it, the algorithm has more information with which to train itself, though the visualization techniques, affected by these confounding factors, would add little value.

There are several key differences between the present our previous work, Leming et al. (2020). While Leming et al. (2020) focused on the use of deep learning on fcMRI, the focus there was on mixed-site data generally and the relative success of classification on three different tasks. In the present work, we elected to focus on sex in the UK BioBank data, rather than multiple datasets, which alleviated previous concerns about site differences from our analysis, and allowed us to focus more on the relative classification of different subgroups within the UK BioBank (i.e., rest/task). To increase the amount of datasets that successfully preprocessed, we also decreased the band count in our wavelet correlation from four to three, since this allowed us to include data in the UK BioBank previously excluded for not having a high enough TR. Differences between our previous analysis and the present analysis, especially the extra data included, may have affected the AUROC on the BioBank data.

Because we wanted to expand our repertoire of visualization methods used to analyze deep learning models after (Leming et al., 2020), we opted to include the occlusion analysis in this paper, and the explicit focus on a-priori networks in the present study was partially motivated by the occlusion method; because of the computational load of occlusion on 300 models, it could only be used practically to test a hypothesis rather than generate those of its own, and so a great deal of time was spent in formulating which brain networks to analyze. Our results show, however, that occlusion may be applied to determine the relative importance of specific edges or networks in subgroups of the overall dataset (e.g., one element of the data may be critical for classifying resting-state data but not matter for task data), offering a significant potential advantage over Grad-CAM.

### 4.2 Neuroscientific interpretations

Four main neuroscientific findings stand out in our results: (1) when classifying sex, the relative AUROC for resting-state data was consistently higher than that for task data by a margin of around 0.12 (Table 1); (2) the within-network edges of the salience network were considered important for characterizing resting-state data (as indicated by both occlusion and CAM results), but not task data (as indicated by occlusion results); (3) edges connecting to all three networks were important in characterizing resting-state fMRI, and notably, even when only considering edges within the networks the p-values for differences between occlusion runs were hardly above 0.05 (Fig. 6); (4) edges connected to the CEN were the only ones that proved important to the classification of both task- and resting-state data together (Fig. 6), even though there was little difference in the distribution of CAM values between them (Fig. 5).

The significantly lower classification accuracy of task data overall compared to resting-state data was consistent both when using complete input data and using partial input data (Table 1). The most straightforward interpretation of this result is that, in task processing, female and male brain function is more similar than it is in the resting-state. Because resting-state brain connectivity varies more than task connectivity (Elton and Gao, 2015), this disparity may also be due to a lower number of distinguishing features.

Explaining the apparent contradiction between our two methods regarding the status of the CEN is complex. Judging from the occlusion results, the CEN is an important network when classifying resting-state data and the only network important in classifying task data, though this is not reflected in the CAMs. Given that these two methods are established visualization methods in ML and a methodical error is unlikely, the takeaway of this contradiction is that these methods are not interchangeable and must be interpreted in their own right, and that the interpretation of specifics in these results ought to be approached cautiously, given the relative novelty of these methods are in their application to neuroscience. Put informally, CAMs show which components of input data the deep learning model pays attention to, while occlusion shows how important a component is to the classification of a specific datapoint; furthermore, because connectivity data is spatially invariant, it may also be the case that our deep learning models consistently focus on the same areas of input, which is reflected in the CAMs, even though this would not be the case for spatially variant photographic data for which CAMs were originally designed (Selvaraju et al., 2017). With this in mind, the similar distribution of CAM values over spatially invariant task- and resting-state input data (see the histograms in Fig. 5) is not surprising since a deep learning model may find a particular edge salient because it might help it to internally subclassify the dataset by resting-state or task. Thus, CAMs may illustrate that a particular edge is important in the overall classification of the model, though not whether it helps in classifying a specific datapoint. With that being said, however, a more thorough study in a pure ML context investigating the mathematical differences between CAM and occlusion results would be necessary.

With regards to the salience network, however, the two methods are more in agreement, since the inner edges of the salience network were clearly the most significant, according to CAMs (Fig. 5). Furthermore, it was the only network with inner edges that proved to be statistically significant to the classification of resting-state data (Fig. 6). This effect may be due, in part, to the particularly salient connection between the left and right amygdala (Fig. 5), which yielded the highest CAM value by far. The DMN is also engaged in sex differences. As can be seen from the middle histogram in Fig. 5, many of its inner edges have a higher class activation than other edges, while excluding it and all edges connected with it had a uniquely negative effect on classification (Fig. 6). What is surprising, however, is that the DMN, which is commonly cited as the marker of resting-state functional connectivity (Raichle et al., 2001) and has previously been implicated in big data sex difference studies (Ritchie et al., 2018) as an area of particular interest, does not stand out from the other two networks studied. While it is not surprising that, in our occlusion tests, the CEN had a greater effect than the DMN in task classification, both tests show that, as stated above, the salience network appears to be more important and have a greater effect on classification accuracy of the resting state. This could be due to a number of factors, such as the use of a priori tests in other studies that specifically account for the DMN, the non-inclusion of subcortical areas in other studies, the inclusion of the critical amygdala connections in the salience network, or other unknown reasons.

## 5 Conclusion

Our results show that the distinction of males and females in resting-state takes into account all of the major brain networks in classification, though they are utilized differently when classifying by resting-state and task. This may be a result of increased variance in resting-state networks over task-based networks, potentially offering the model a larger set of distinguishing markers. When only considering the emotional faces recognition task of the UK Biobank, areas connecting to the DMN and, more so, the CEN showed significantly altered function, while function of the salience network was not different enough to significantly aid in single-subject classification (Fig. 6). Methodologically, we have demonstrated the applicability and limitations of two different deep learning visualization methods to brain network data, as well as deep learning’s applicability to big data in a scientific field.

## CRediT authorship contribution statement

**Matthew Leming:** Conceptualization, Data curation, Formal analysis, Investigation, Methodology, Project administration, Software, Visualization, Writing – original draft, Writing – review & editing. **John Suckling:** Funding acquisition, Methodology, Project administration, Resources, Supervision, Validation, Writing – review & editing.
